# Testing gene set enrichment for subset of genes: Sub-GSE

**DOI:** 10.1186/1471-2105-9-362

**Published:** 2008-09-02

**Authors:** Xiting Yan, Fengzhu Sun

**Affiliations:** 1 Molecular and Computational Biology Program, Department of Biological Sciences, University of Southern California, Los Angeles, CA 90089-2910, USA

## Abstract

**Background:**

Many methods have been developed to test the enrichment of genes related to certain phenotypes or cell states in gene sets. These approaches usually combine gene expression data with functionally related gene sets as defined in databases such as GeneOntology (GO), KEGG, or BioCarta. The results based on gene set analysis are generally more biologically interpretable, accurate and robust than the results based on individual gene analysis. However, while most available methods for gene set enrichment analysis test the enrichment of the entire gene set, it is more likely that only a subset of the genes in the gene set may be related to the phenotypes of interest.

**Results:**

In this paper, we develop a novel method, termed Sub-GSE, which measures the enrichment of a predefined gene set, or pathway, by testing its subsets. The application of Sub-GSE to two simulated and two real datasets shows Sub-GSE to be more sensitive than previous methods, such as GSEA, GSA, and SigPath, in detecting gene sets assiated with a phenotype of interest. This is particularly true for cases in which only a fraction of the genes in the gene set are associated with the phenotypes. Furthermore, the application of Sub-GSE to two real data sets demonstrates that it can detect more biologically meaningful gene sets than GSEA.

**Conclusion:**

We developed a new method to measure the gene set enrichment. Applications to two simulated datasets and two real datasets show that this method is sensitive to the associations between gene sets and phenotype. The program Sub-GSE can be downloaded from .

## Background

Genome-wide gene expression profiling using microarray technologies has been ubiquitously used in biological research. An important problem is to identify gene sets that are significantly changed under a certain treatment (for example, two different cell lines or tissues or the same cell line under different conditions). A gene set is basically a group of genes with related functions, e.g., genes in a biological process or in the same complex. There are a variety of ways by which genes, and, ultimately, gene sets may be defined. For example, gene sets can be defined according to the information provided by several databases, such as GeneOntology [1], KEGG [2], Biocarta , and Pfam [3]. Gene sets may also be defined by cytogenetic bands, by region of genomic sequence or by establishing the functional relationships among them. Importantly, by using a gene set-based approach, a high power can potentially be achieved for detecting differentially expressed gene sets by integrating expression changes of genes inside the same gene set, even when the expression changes of individual genes are modest.

Moreover, because the gene sets have already been annotated by their common functions in the databases, the biological interpretation for a given list of significant gene sets will also be clear. At least one study [4] showed that using such gene set-based approaches did increase the congruence of the identified gene sets between different data sets addressing the same biological problem. To detect differentially expressed gene sets, several methods have been proposed, which can be roughly categorized into three groups.

The first group identifies a list of significant differentially expressed genes (DEGs) using individual gene analysis methods, and then examines the enrichment of gene sets within this gene list using different statistical tests, such as the binomial test, Fisher's exact test, or the hypergeometric test [5-11]. Khatri and Draghici [12] compared fourteen different methods within this group. Each of these methods is easy to implement, but flawed by 1) sensitivity to the cutoff value for defining the list of significant DEGs, 2) non-consideration of the relative position of genes inside the significant DEG list, and 3) assumption of independence between the genes, which may make the resulting p-value misleading.

The second group of methods does not depend on the predefined DEG list. Instead, these methods calculate a gene-specific statistic, known as the "local" statistic, which measures the strength of association between the gene expression and the phenotype for each gene. A "global" statistic for a gene set is then constructed as a function of the local statistic for each gene in it. The significance of the global statistic is assessed by permutation test, and different methods arrive at this assessment in different ways [13-21]. In contrast to calculating a gene-specific statistic, the third group of methods directly combines the expression levels of all genes in the gene sets and they are represented as gene set-specific features. These features are then compared between the treatment and the control groups to identify significantly affected gene sets [22-27]. Some methods also integrated the interaction information between genes in the gene sets [28-31].

The available methods generally tested the association of all the genes in a gene set with the phenotype. In reality, however, it is more likely that only genes in a subset of the gene set of interest are associated with the phenotype. Three possible factors may explain this. First, since the function annotation defined in the available databases, such as GO, KEGG, and Biocarta, are incomplete, or even erroneous, some of the genes in a gene set of interest may not truly belong to the set. Second, the gene sets are sometimes defined according to the genomic regions of the genes. Thus, the expression of the genes in the same set may not coordinate with each other. For example, although a group of gene sets has been defined by the cytogenetic bands on human chromosomes [14], the expression of genes on the same cytogenetic bands do not necessarily correlate with each other. The result is that only a subset of genes in a given cytogenetic band will then be correlated with a phenotype. Third, even if all the genes in the gene set have the same function, or belong to the same complex, it is possible that only genes in one branch of the pathway are associated with the phenotype. The cumulative effect of these considerations strongly suggests that the currently available methods for gene set enrichment analysis may not be powerful enough to detect the association of a given gene set with a phenotype, particularly in the case where only a subset of the genes is associated with the phenotype.

In this paper, therefore, we extend a set-association strategy for genetic polymorphism association studies developed by [32] to a set-enrichment analysis. In so doing, we want to test the null hypothesis that no subsets of genes in the gene set are associated with the phenotype. We refer to the resulting method as Gene Set Enrichment by testing Subset association, or Sub-GSE. Using two simulated data sets, we first show that Sub-GSE has higher sensitivity in identifying gene sets associated with a phenotype compared to GSEA, SigPath, and GSA, when only a fraction of the genes are associated with the phenotype. Next, we apply Sub-GSE to two real data sets. One involves gene expression data related to gender and the other identified functional gene sets related to p53 mutation status. For the first dataset, Sub-GSE identified cytogenetic bands Xp22 as significantly associated with gender, while GSEA failed to detect this association. For the second dataset, Sub-GSE identified several novel functional gene sets, including DNA damage genes, cell cycle checkpoints genes and programmed cell death genes that are associated with p53 mutation status and that were also not detected by GSEA. Overall, this method provides a complementary approach for identifying gene sets associated with a phenotype, especially when only a subset of genes in a gene set is associated with the phenotype.

## Results and discussion

In this section, we first give a brief overview of our method. Second, we apply Sub-GSE, GSEA, SigPathway, and GSA to two simulated data sets and compare their performance. Third, we apply Sub-GSE to two real datasets: one related to gender and the other related to p53 mutation status. We also show some new biological findings related to the two real data sets using Sub-GSE.

### Outline of the method

To assess the enrichment of a given gene set, we construct a statistical hypothesis testing model. The null hypothesis is that no subsets of genes in the given gene set are associated with the phenotype.

#### Defining the "strict subsets"

Given the fact that the number of all subsets of a gene set increases exponentially with the number of genes in the gene set, it is impractical and less powerful to test every subset of the gene set. Therefore, we define the "strict subsets" to only those subsets that are more likely to be related with the phenotype. To define the "strict subsets" of a gene set, we first calculate the association strength between the gene expression and the phenotype for each gene in the gene set. Depending on the measurement levels of the phenotypic data, we calculate the absolute t-statistics for comparing the mean gene expression levels for binary phenotypic data, Kruskal-Wallis statistics for comparing the mean expression levels of different groups for discrete phenotypic data, and the absolute Pearson correlation coefficient between the gene expression levels and the phenotype for continuous data. All genes are sorted in decreasing order of the association strength measures. The "strict subsets" are defined to include genes up to each position from the top to the bottom in the ranked gene list, which are most strongly associated with the phenotype. That is to say, for each position in the ranked gene list, we define a strict subset that includes all the genes that are ranked higher than this position. Thus, if there are *n *genes in the gene set, there will be *n *"strict subsets" among which the *i*-th subset contains the top *i *genes in the ranked gene list according to the association strengths. In this way, the number of subsets to be tested increases linearly with the number of genes in the gene set. Since the strict subsets includes the genes that are most associated with the phenotype, we expect them to be more probable to be related with the phenotype. The strict subsets are defined to be contiguous to include as many as possible subsets that are expected to be more likely related with the phenotype. However, the method we propose here cannot detect the gene sets in which individual genes are not associated with the phenotype but they can interact with each other to affect the phenotype. To overcome the problem that the "strict subsets" contain too few genes, we add a tuning parameter to control the sizes of the "strict subsets". Throughout the paper, we set this tuning parameter to be 5 which means the "strict subsets" are required to contain at least 5 genes. The cutoff for the set size of the strict subsets is set to be 5 so that the method is not too sensitive to detect gene set which has only one gene strongly correlated with the phenotype. There are other ways of deciding the cutoff of the set size as discussed in the Conclusions section.

#### Testing Statistic

The hypothesis testing statistic is calculated in three steps. First, for each "strict subset", we calculate the average association strength across all member genes, which is also called the local set association statistic *T*. Second, the statistical significance (raw p-value) of the local set association statistic *T *for each "strict subset" is calculated by permuting the phenotypes of the individuals. Finally, the minimum raw p-value among all the "strict subsets" is evaluated and taken as the hypothesis testing statistic. If there is any strict subset related with the phenotype, the minimum p-value will be significantly small.

#### Significance Assessment

To assess the significance of the minimum p value, nested permutation is needed since we do not know the distribution of *T *under the null hypothesis However, nested permutation is computation intensive. Fortunately, previous work [33] has shown that a single set of permutation is sufficient to accomplish the significance assessment. For the permutation, we decide to permute the phenotypic data and keep the gene expression data intact due to the criticism on gene-based permutation which assumes the independence between genes [16].

The phenotypic data is permuted for *N *times. After each permutation, the "strict subsets" are re-defined according to the newly calculated association strengths using the permuted phenotypic data. The strict subsets are defined in the same way as we did with the observed data including the threshold of set size.

The only difference is that the phenotypic data is changed. By comparing the set association statistic *T *from the observed data and those from the permuted data, raw p values can be calculated for all the observed "strict subsets" and thus the observed *P*_*min *_is obtained. To estimate the distribution of *P*_*min *_under the null hypothesis, as classic permutation does, we replace the observed phenotypic data with every permuted phenotypic data and compare the set association statistic with those from all the other *N *- 1 permutations. In other words, we repeat exactly the same procedure to obtain the minimum raw p values for every permuted data. Finally, the significance of the gene set will be the percentage of permutations that result in minimum raw p values smaller than the observed *P*_*min*_. If there are more than one given gene set, multiple testing correction can be done using any multiple testing correction method. In this paper, we use the QVALUE R package [34] to calculate the q-values for the two biological data sets so that the results by Sub-GSE are comparable with other gene set enrichment analysis methods.

### Simulation Studies

We first elucidate that the p-values do not depend on the size of the gene set and the p-value has a uniform distribution in [0,1] under the null hypothesis. To achieve these objectives, we simulate a data set where all the gene sets have different set sizes and no gene sets are related to the phenotype. The simulation is implemented in the following steps:

1. Generate 100 gene sets whose sizes are 5,6,7,8,...,104. The total number of genes is 5450;

2. The gene expression levels in 100 samples for each gene are generated from a standard normal distribution. Different genes are independent of each other. Different samples are also independent of each other;

3. Generate the phenotypic data from another independent standard normal distribution in 100 samples;

4. Repeat steps 1–3 for 100 times;

In total, the simulation generates 100 data sets that have gene expression data and a corresponding phenotypic data. We apply Sub-GSE to the 100 data sets separately.

**Figure 1:**
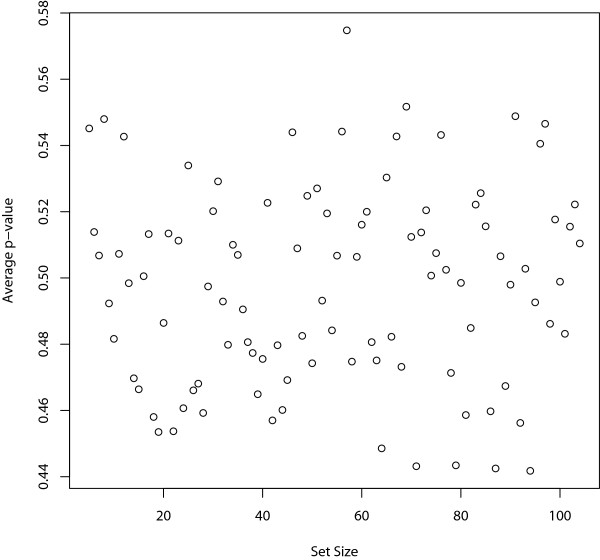
**Effect of set size on the p-values**. The average p-values of the 100 gene sets across the 100 different data sets are plotted against their corresponding set sizes.

First, since the gene sets have different sizes, we plot the average p-values of all the gene sets across the 100 different data sets against their set sizes to see whether the gene set size affects the significance level. Figure 1 shows that the set size does not affect the p-values.

**Figure 2:**
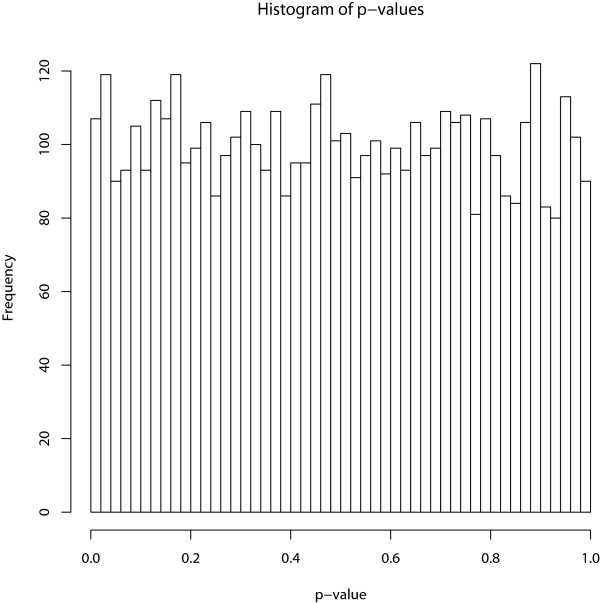
**The distribution of p-values under the null hypothesis of no association**. The histogram of the p-values under the null hypothesis of no association between the gene sets and the phenotype.

Second, the phenotypic data is independent of the expression levels of all the genes. Therefore, Sub-GSE should not detect any significant gene sets. In Figure 2, the histogram of all the p-values of the 100 gene sets from the 100 data sets is shown. The histogram illustrates that the p-values from the Sub-GSE have a uniform distribution for gene sets that are not related to the phenotype, which is consistent with the theoretical uniform distribution under the null hypothesis.

#### Simulation 1

We first evaluate the performance of Sub-GSE using simulated data in which gene expression profiles with different correlations within the gene set are generated. The expression profiles for 1000 genes in 100 samples are simulated. The genes are divided into 50 non-overlapping gene sets with 20 genes in each. The gene expression profiles for the 100 samples represent 100 independent vectors of random variables generated from a multivariate normal distribution. The multivariate normal distribution has 1000 dimensions corresponding to the 1000 genes. The mean is a vector of 1000 zeroes, and the variance of the expression levels of each gene is 1. To simulate the dependence between genes, we randomly select a certain percentage of correlated genes (PCG) = (0%, 10%, ⋯, 90%) in each gene set and let the correlation coefficient between any two of them be *ρ *= 0, 0.1, 0.2, ⋯, 0.9. The remaining genes are independent of each other and those that are chosen. We use this simulation strategy based on the following considerations. The chosen genes in the gene set correspond to those in the same complex or pathway; thus, their expression profiles are correlated. Also, since the remaining genes represent those not belonging to the group, they are more likely to be independent of the chosen genes and each other.

If a given gene is among those that are chosen, we use its expression levels as the phenotype. The rationale of this step is to determine if our Sub-GSE method can identify the gene set to which this particular gene belongs. We repeat this process for all the chosen genes. Thus, we have a total of 1000 × *PCG *different phenotypic data. To avoid the problem where a gene has exactly the same expression profile as the phenotype, we eliminate the gene's expression profile from the expression data if it is used as the phenotype.

**Figure 3:**
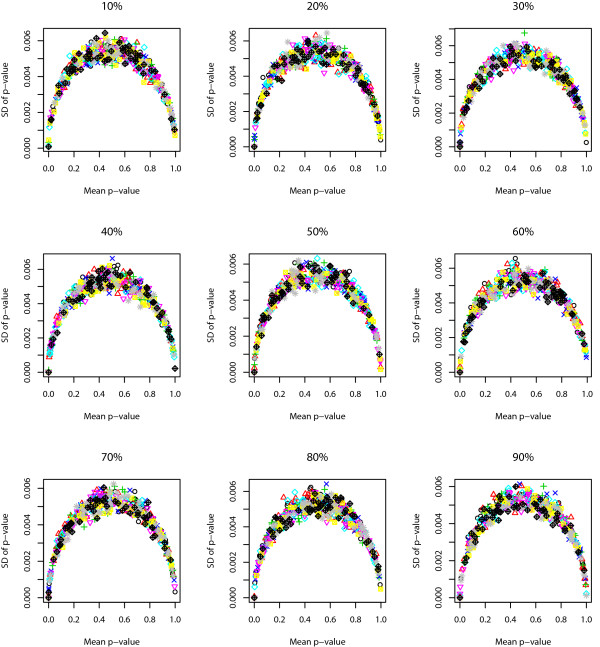
**The robustness of Sub-GSE based on simulation 1**. The standard deviation of the p-values across the 100 runs of Sub-GSE on simulation 1 is plotted against the average p-values for all the gene sets.

We use the following approach to study the robustness of Sub-GSE. For each given correlation coefficient and PCG, we randomly choose one of the simulated phenotypic data and the corresponding gene expression data. Sub-GSE is applied to the chosen data set for 100 times. The standard deviations of the p-values across the 100 different runs are plotted against the average p-values for all the gene sets in Figure 3. The figure shows that the standard deviation of the p-value for the same gene set is smaller than 0.006 and even smaller when the p-value is close to either 1 or 0. The closer the p-value is to 0 or 1, the smaller the standard deviation is. The maximum standard deviation is achieved when the average p-value is around 0.5.

**Figure 4:**
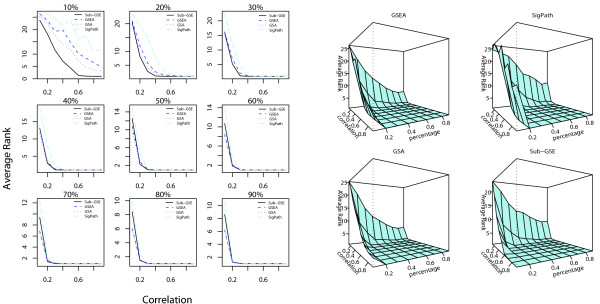
**Comparison results of the different tests based on simulation 1**. The average ranks of the gene set related to the phenotype for Sub-GSE, GSEA, GSA, and SigPath for different percentages of correlated genes (*PCG*) and correlation coefficients within the chosen genes. The left panel compares the average ranks in 2-D plots in which each subplot corresponds to one value of *PCG*. For a given *PCG*, the average ranks from the four methods are plotted against the correlation coefficient between the correlated genes. The right panel shows the average rank versus the percentage of correlated genes and correlation coefficient in a 3-D plot.

For each given pair of percentage of correlated genes (*PCG*) and correlation coefficient, we apply all four methods, Sub-GSE, GSEA, GSA, and SigPath, to the corresponding data. All the gene sets are ranked in an increasing order of their q-values so more significant gene sets have smaller rank. The rank of the gene set, some of whose member genes are correlated with the phenotypic data, is extracted to evaluate the performance of the methods. Figure 4 shows the average rank of the gene set related to the phenotype for different combinations of *PCG *and correlation coefficient.

First, as seen in the left panel in Figure 4, for small *PCG *= 10%, 20% and 30%, the average rank of the gene set related to the phenotype based on Sub-GSE is always the lowest, irrespective of the coefficient value. On the other hand, for large *PCG*, the performance of Sub-GSE is similar or slightly worse than GSEA and GSA for small correlation. The right panel in Figure 4 confirms this because the average rank of the gene set related to the phenotype based on Sub-GSE decreases much faster than those for the other methods when *PCG *is small. The results of Figure 4 can be explained as follows. When *PCG *is low, only a small fraction of the genes in the target gene set are correlated with the phenotype. GSEA, GSA, and SigPath cannot distinguish the target gene set from the other gene sets since these methods consider all the genes in the gene set of interest in their statistics. In contrast, Sub-GSE incrementally tests each strict set and chooses the smallest p-value across all the strict sets as a test statistic, thus making the test more powerful.

Second, across different combinations of *PCG *and correlation coefficient, we find that GSA and GSEA achieve similar results. Both GSA and GSEA use t-statistics to obtain the ranking list of genes. For applications in this article, the only diference between them is that GSA restandardizes the statistics before the permutation to reduce the effect of correlation between genes. However, in both panels of Figure 4, the average ranks of the target gene set by GSEA and GSA are quite similar, especially when the *PCG *is high. Consequently, restandardization in GSA does not seem to be very efficient in this simulation study, especially when there are many correlated genes.

Third, to show the sensitivity and specificity of Sub-GSE, we need a group of gene sets that are related with the phenotype. Therefore, we do another set of simulations similar as simulation 1. The detailed descriptions of the simulation and the resulting ROC curves can be found in the supplementary materials [see Additional file 1]. The results show that the higher the PCG and the correlation coefficient are, the higher the AUC score is. Once the correlation coefficient is higher than 0.4, the AUC score is higher than 0.85 no matter what the PCG is. When PCG is higher than 0.5, the AUC score can be higher than 0.75 regardless of the correlation coefficient.

#### Simulation 2

In reality, most phenotypes are the joint effect of multiple genes, probably from multiple pathways. Therefore, we also simulate a more realistic case where the phenotypes are assumed to be a complex function of expression levels from two gene sets. As in simulation 1, we again consider 1000 genes divided equally into 50 non-overlapping gene sets of 20 genes in each. For fixed *PCG *and *ρ*,

1. Simulate the expression profiles of the 1000 genes as in the first simulation for 100 individuals;

2. Choose two gene sets *K*_1 _and *K*_2 _from the 50 gene sets. Let *SK*_1 _and *SK*_2 _be the correlated genes in *K*_1 _and *K*_2_, respectively. Define the phenotype for the *j*-th individual as

$$y_{j}=1+\sum_{i\in SK_{1}}e_{ij}^{2}+\sum_{i\in SK_{2}}e_{ij}^{2}+\varepsilon_{j},$$

where *ϵ*_*j *_has a normal distribution with mean 0 and variance 0.25.

3. Analyze the data using GSEA, SigPath, GSA, and Sub-GSE to rank the gene sets. Rank all the gene sets in increasing order of their q-values.

4. Repeat steps 1–3 100 times to assess the performance of the different analytic methods by the effects of the different gene expression data.

**Figure 5:**
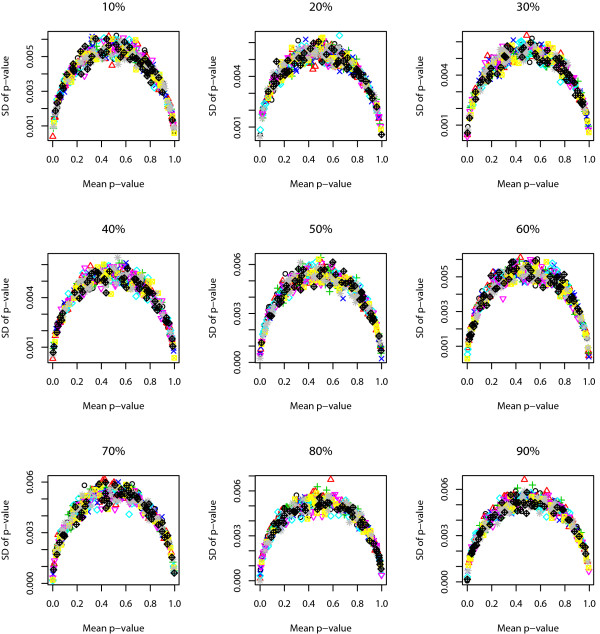
**The robustness of Sub-GSE based on simulation 2**. The standard deviation of the p-values across the 100 runs of Sub-GSE on simulation 2 is plotted against the average p-values for all the gene sets.

We study the robustness of Sub-GSE as follows. Similar to the process in simulation 1, for each given correlation coefficient and PCG, we randomly choose a phenotypic data and the corresponding gene expression data. Sub-GSE is applied to the chosen data set for 100 times. The standard deviation of the p-values across the 100 runs for all the gene sets is plotted against the average p-values in Figure 5. Again, the standard deviation of the p-values across different runs for each gene set is smaller than 0.006. The closer the average p-value is to either 0 or 1, the smaller the standard deviation is. The maximum standard deviation is achieved when the average p-value is around 0.5.

**Figure 6:**
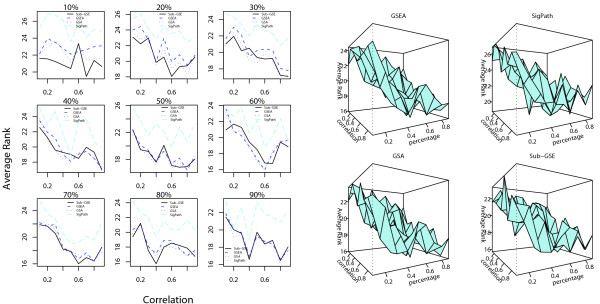
**Comparison results of the different tests based on simulation 2**. The average ranks of the target gene sets by Sub-GSE, GSEA, GSA and SigPath for different percentages of correlated genes and correlation coefficients in Simulation II. The left panel compares the average ranks in 2-D plots in which each subplot corresponds to one value of *PCG*. For a given *PCG*, the average ranks of the target sets from the four methods are plotted against the correlation coefficient between the correlated genes. The right panel shows the average ranks of the target sets versus the *PCG *and correlation coefficient in a 3-D plot. The four cubes correspond to Sub-GSE, GSEA, GSA and SigPath.

For this simulation study, we again apply the four different methods to prioritize the gene sets as in the first simulation study and calculate the average rank of the two target gene sets. The results can be found in Figure 6.

As shown in Figure 6, the average ranks of the target gene sets based on all the methods are relatively high. This could result from the involvement of two different gene sets when simulating the phenotypic data and the fact that the phenotypic data are the sum of the squared expression levels of correlated genes. Another potential complicating factor is that the phenotype includes a noise in addition to the function of the gene expression levels of the component genes. All these facts can weaken the correlation between the phenotypic data and the gene expression profile of individual genes inside the true gene sets. Despite these problems, Sub-GSE performs relatively well compared to the other three methods, especially when *PCG *is low. When *PCG *is high, the performances of Sub-GSE are close to those of GSEA and GSA since both GSEA and GSA consider all the genes inside the gene sets. Again, the performances of GSEA and GSA are similar.

### Male Vs. Female Lymphoblastoid Cells

We also apply Sub-GSE to two real data sets from [14]. The first data set measured the mRNA expression profiles from lymphoblastoid cells derived from 15 males and 17 females using Affymetrix U133A chip. The gender of the individuals represents the corresponding phenotypic data. The gene sets are chosen as the cytogenetic sets (C1, 319 gene sets) and the functional gene sets (C2, 522 gene sets) defined in [14]. The cytogenetic sets contain 24 gene sets, one for each of the 24 human chromosomes, and 295 gene sets corresponding to cytogenetic bands along the chromosomes. The functional sets include 472 gene sets containing genes whose products are involved in specific metabolic and signaling pathways, as reported in eight publicly available, manually curated databases, and 50 gene sets containing genes co-expressed in response to genetic and chemical perturbations, as reported in various experimental studies (see supporting text in [14] for details). We apply Sub-GSE, GSEA, and SigPath to these two types of gene sets independently with the objective of identifying the cytogenetic regions that are differentially expressed between males and females and the functional gene sets related to sex distinction, respectively.

First, we apply Sub-GSE to investigate the enrichment of cytogenetic gene sets (C1). As expected, the three most significant cytogenetic bands are chrY, chrYp11 and chrYq11 which all have a q-value of 0 and are the only three cytogenetic bands from chromosome Y in gene sets C1. They are also the only three significant gene sets in C1 by GSEA (FDR < 0.2) and SigPath (max q-value < 0.2). Besides these expected bands on chromosome Y, other bands that are ranked as the top 7 among all the gene sets by Sub-GSE, GSEA and SigPath are listed in Table 1. As seen from the lists, Sub-GSE is sensitive enough to identify cytogenetic bands on both chromosomes X and Y at the q-value threshold of 0.20. On the contrary, neither GSEA nor SigPath is able to detect any bands on chromosome X at the FDR threshold of 0.20. Again, this result shows the sensitivity of Sub-GSE.

**Table 1:** Comparison of the top 7 cytogenetic bands related to gender detected by different methods.

Sub-GSE		GSEA		sigPath	
set names	q-value	set names	FDR	set names	max q-value

chrY	0	chrY	0	chrY	0
chrYq11	0	chrYq11	0.000801147	chrYq11	0
chrYp11	0	chrYp11	0.000811309	chrYp11	0
chrXp22	0.147	chrYp11_Xp22	0.25508726	chrYp11_Xp22	0.315700257
chrX	0.294	chr11p12	0.31999215	chr1q22	0.990782566
chrXp11	0.686	chr6q24	0.6108403	chr20p11	0.991916264
chr15q11	0.924	chr5p14	0.6131663	chr15q21	0.991916264

The top 7 gene sets by Sub-GSE, GSEA and sigPath and their corresponding p-values, FDR and max q-values. The cytogenetic bands chrYp11_Xp22 was named chrYp22 in the original gene set data which include 8 genes that are on both chrYp11 and chrXp22.

Second, we apply Sub-GSE to investigate the enrichment of functional gene sets (C2). Both Sub-GSE and GSEA detect three significant gene sets whose significance levels are listed in Table 2: testis-related genes, genes that escape X inactivation, and female reproductive tissue-expressed genes. The q-values of all the other gene sets are larger than 0.9 by Sub-GSE. Hence, in this dataset, the results by Sub-GSE are roughly the same as those achieved by GSEA.

**Table 2:** Comparison of the significant functional gene sets related to gender by Sub-GSE and GSEA.

Set Name	Sub-GSE (q-value)	GSEA (FDR)
Testis related genes	<0.001	0.012
Genes that escape X inactivation	0.165	<0.001
Female reproductive tissue expressed genes	0.165	0.045

The top 3 functional gene sets and their corresponding q-values and FDR rates by Sub-GSE and GSEA.

### P53 Status in Cancer Cell Lines

**Figure 7:**
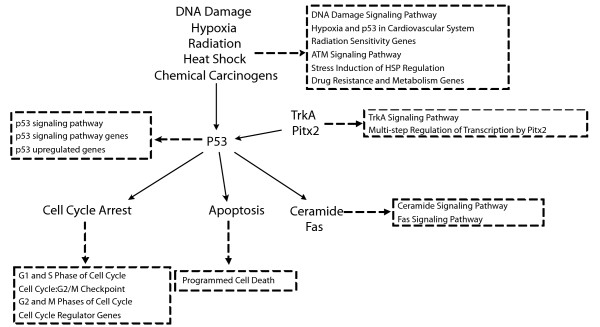
**Relationship between the significant gene sets and p53**. Relationship between significant gene sets and p53. Solid arrows describe the interactions between p53 and different biological processes or genes. Dashed arrows show the definition of those gene sets in the corresponding dashed rectangle. The solid arrows are constructed based on previous works in [35-40].

The second real data set corresponds to the gene expression data and phenotypic data related to p53 mutation status from [14]. The objective of this study is to identify novel targets of the transcription factor p53. The p53 mutation status gene expression data examined the gene expression patterns from the NCI-60 collection of cancer cell lines. The expression profiles were measured using Affymetrix U95Av2 chips. The mutational status of the p53 gene had been reported for 50 of the NCI-60 cell lines, with 17 being classified as normal and 33 as carrying mutations in the gene. We take the gene expression profiles of these 50 cell lines as the expression data and the vector of binary indicators of the mutational patterns (normal or mutated) as the corresponding phenotypic data. For the gene set data, we only use the functional sets (C2, 522 gene sets) in [14], which was already described in the application noted above. Functional sets that have a q-value smaller than or equal to 0.03 by Sub-GSE are extracted and listed in Table 3 together with their q-values. Comparing the list of significant gene sets by Sub-GSE and GSEA [14], we can see that Sub-GSE obtains more significant gene sets than GSEA does, which again shows the sensitivity of Sub-GSE. The relationship between the identified gene sets by Sub-GSE (Table 3) and p53 is illustrated in Figure 7. Basically, all these gene sets are significantly enriched in genes that are differentially expressed in p53 mutants versus those without p53 mutations. Therefore, the identified gene sets by Sub-GSE are potentially those regulated by p53. According to the definitions of these gene sets, as shown in Figure 7, we can roughly divide them into three groups.

**Table 3:** Significant functional gene sets related to p53 mutational status by Sub-GSE.

Set Name	q-value
Hypoxia and p53 in the Cardiovascular system	<0.001
G1 and S Phases of the Cell Cycle	<0.001
p53 Signaling Pathway (p53 Pathway)	0.022
TrkA Signaling Pathway	0.022
P53 Upregulated Genes	0.022
p53 Signaling Pathway Genes(p53_signalling)	0.029
Cell Cycle: G2/M Checkpoint	0.029
G2 and M Phases of the Cell Cycle	0.029
Programmed Cell Death	0.029
DNA Damage Signaling Pathway	0.029
Radiation Sensitivity Genes	0.029
Cell Cycle Regulator Genes	0.029
ATM Signaling Pathway	0.029
Ceramide Signaling Pathway	0.029
Drug Resistance and Metabolism Genes	0.029
Stress Induction of HSP Regulation	0.029
Multi-step Regulation of Transcription by Pitx2	0.029
Fas Signaling Pathway	0.029

Significant functional gene sets (q-value ≤ 0.03) detected in 50 of the NCI-60 cell lines. The q-values are calculated by Sub-GSE.

The first group includes gene sets that are directly regulated or affected by p53, including the "p53 signaling pathway", "p53 signaling pathway genes", and "p53 upregulated genes". This group of gene sets was detected by both Sub-GSE and GSEA [14] (FDR < 0.015).

The second group contains gene sets that are "downstream" of p53. These gene sets can either be induced or inhibited by p53 [35-38]. For example, it is well known that p53 induces cell cycle arrest during the G1/S phase and the G2/M phase checkpoint [36]. By itself, p53 can activate an important death receptor, Fas, which triggers the "Fas Signaling Pathway" and thus leads to apoptosis [38]. It is also well known that p53 functions "upstream" of ceramide in response to genotoxic stress [37].

The third group includes gene sets related to the "upstream" biological processes or genes for p53. These "upstream" biological processes, such as DNA damage caused by radiation or chemical carcinogens, for example, pass the DNA damage signal down to p53 and further induce some of the "downstream" pathways. Two genes, TrkA and Pitx2, are known to affect apoptosis through regulation of p53 [39,40]. The gene sets related to these "upstream" biological processes actually include genes related to those "downstream" biological processes in the second group.

In this dataset, Sub-GSE not only detects the gene sets identified by GSEA [14], but also detects more novel gene sets related to p53. Previous studies from the literature support the findings in that all the significant gene sets identified by Sub-GSE are related to p53, as shown in Figure 7.

## Conclusion

To summarize, we have developed a method, called Sub-GSE, to identify gene sets that are associated with a phenotype by testing the association between the strict subsets of genes and the phenotype. In many applications, it is very likely that only a subset of genes in a gene set of interest is associated with the phenotype. However, since currently available methods for gene set enrichment analysis usually test the association of all the genes in a gene set with the phenotype, the power of these methods is correspondingly reduced. In contrast, Sub-GSE is based on the idea of set-association approach first proposed by [32] and it incrementally tests the association of "strict subsets" with the phenotype. The strict subsets contain the genes having the top association strength of individual genes with the phenotype. We first study the performance of Sub-GSE and compare it with three widely used methods for gene set enrichment analysis: GSEA, GSA, and SigPath. Our simulations show that Sub-GSE outperforms GSEA, GSA, and SigPath in prioritizing gene sets associated with a phenotype when the fraction of genes associated with the phenotype is relatively small. On the other hand, these four methods all achieve similar results when the fraction of associated genes is large. When applied to two real data sets, Sub-GSE is shown to detect more biologically meaningful gene sets than GSEA. For example, Sub-GSE identified cytogenetic band Xp22 as significantly associated with gender (q-value < 0.20), while neither GSEA nor SigPath identified them as significant at a FDR < 0.20. Similarly, Sub-GSE identified many gene sets including, for instance, DNA damage genes, cell cycle checkpoints genes and programmed cell death genes, as significantly associated with p53 mutation status. These were not identified by GSEA. This evidence supports the high sensitivity of Sub-GSE. Most of the detected gene sets have supports from previous studies for the association between them and the p53 mutations.

Usually a large number of sets will be detected as significant for most tests of gene enrichment analysis. Since Sub-GSE is more sensitive in detecting significant gene sets than other tests for gene set enrichment analysis, we expect that many more gene sets will be identified as significant. This may reflect biological reality instead of statistical artifacts. For example, cancer can affect a large number of genes and gene categories. By studying the GO relationship among the significant gene sets, the more specific significant GO categories may represent the real underlying affected function categories.

The advantages of Sub-GSE over other approaches for testing gene set enrichment are most evident when only a fraction of the genes in the gene set of interest are associated with the phenotype. If we believe that most genes in a gene set of interest are associated with the phenotype, other approaches, including GSEA, GSA, and SigPath, may perform better than Sub-GSE. Under this scenario, the use of the minimum p-value across all the strict subsets as a test statistic, which is done in Sub-GSE, would result in the introduction of more noise. It is possible that the minimal p-value may be achieved for some subsets of the gene set of interest, making Sub-GSE less powerful. The results of our simulations are consistent with this observation. On the other hand, our simulations also showed that the performance of Sub-GSE is only marginally worse than the other approaches under the conditions noted above. We do not claim that Sub-GSE is always better than GSEA, GSA, or SigPath. Instead, Sub-GSE complements other approaches for gene set enrichment analysis when the fraction of associated genes is relatively small.

The speed of Sub-GSE is determined by the number of gene sets and the number of genes inside each gene set. To give an example of the running time, we download the gene expression data with accession number GSE5081 from NCBI  which hybridized total RNAs from gastric biopsy specimens of patients with Helicobacter pylori positive (HP+) and Helicobacter pylori negative (HP-) antrum erosions (ER+), and the corresponding, adjacent normal mucosae (ER-). The gene expression data includes 54675 probes and 32 samples. HP+ and HP- are treated as the phenotype. Mappings between the probes and GO categories are from the R package  named "hgu133plus2". All the probes are mapped to 8310 GO categories in total. We run Sub-GSE on this data set using a computer with Pentium 4 CPU 3.60 GHz/3.59 GHz, 1.00 GB of RAM. It took 12.7 hours when 1000 permutations are required and the strict set size threshold is 1.

Usually we need to simultaneously test the association of a large number of gene sets with the phenotype. For each gene set, we can use Sub-GSE to test the association of the gene set with the phenotype to obtain a p-value. We have shown in the "Results" section that the p-value is uniformly distributed under the null hypothesis that no subset is associated with the phenotype. When we test for a large number of gene sets, the issue of multiple testing is of concern. To solve this problem, conventional methods such as Bonferroni correction can be used. However, Bonferroni correction is too conservative in most situations. Another currently widely used method dealing with multiple testing is to control false discovery rate (FDR) as implemented in the software package QVALUE [34]. For the QVALUE package to work well, the p-values for all the gene sets need to be weakly dependent. When the sizes of the gene sets are relatively small compared to the total number of genes, we expect that the p-values to be weakly dependent since the genes usually form modules and genes from different modules are more likely to be independent. When these assumptions are in doubt, we can use the p-values obtained from Sub-GSE to indicate the statistical significance of the gene sets.

There are two options in Sub-GSE: the minimal size for the strict sets and the statistic to measure the association strength between gene expression profiles and the phenotype. We set the tuning parameter *c *to be the minimal size of the strict subsets on which to test. Parameter *c *can control the sensitivity and the specificity of Sub-GSE, thus having a significant effect on its performance. Generally, the sensitivity of Sub-GSE decreases and the specificity increases as *c *increases. Therefore, the choice of *c *should depend on the balance between sensitivity and specificity. Although we set *c *= 5 in this paper, which restricts the minimal size for the strict sets, we can, instead, require that the minimal size of the strict sets depend on the size of the gene set of interest. For example, one could consider the subsets of genes that cover at least 10% of the given genes inside each gene set. Since the minimal set size of the subset may be different for different gene sets, the effects of this type of restriction need to be further studied. The other Sub-GSE option involves the statistic used to measure the association strength between gene expression profiles and the phenotype. In this paper, we use t-statistics, Kruskal-Wallis statistics, and Pearson's correlation to evaluate the association strength between the gene expression profiles and discrete, categorical, and quantitative phenotypes, respectively. Other statistics can also be applied. The power of Sub-GSE to detect enriched gene sets for different types of statistics also needs to be further studied.

It is well known that genes in the same pathway or complex tend to be correlated. A natural question is whether it is better first to do principal component analysis (PCA) and then apply Sub-GSE to the principal components. We implemented this idea and found the approach less powerful than the method implemented in this paper. A potential explanation is that the expression profiles of the genes in the gene sets among the cases and controls do not satisfy the normality assumption making the PCA approach less powerful. More studies are needed to see under what conditions the combination of PCA and Sub-GSE is more powerful than Sub-GSE alone.

## Methods

### Association strength measures for individual genes

For a given gene, suppose the gene expression levels measured in the experiment are (*e*_1_, *e*_2_, ⋯, *e*_*m*_), where *m *is the number of samples. The corresponding phenotypic data for the *m *samples are denoted as *C *= (*c*_1_, *c*_2_,⋯, *c*_*m*_). Depending on the measurement levels of *C*, we measure the association strength between the gene expression and phenotype by the absolute value of t-statistic, Kruskal-Wallis statistic or Pearson correlation coefficient for binary, discrete or continuous phenotypic data, respectively.

### Local association statistic for each strict subset and the global statistic for the gene set

Suppose the given gene set has a total of *s *genes and the sorted association strength measures are *a*_1 _≥ *a*_2 _≥ ⋯ ≥ *a*_*s*_. According to the definition of the strict subsets, the *i*-th strict subset include genes that correspond to (*a*_1_, *a*_2_,⋯, *a*_*i*_). The local association statistic for the strict subset *i *is defined as

$$T_{i}=\frac{\sum_{j=1}^{i}a_{j}}{i}.$$

We use the permutation test described below to define a p-value, *p*_*i *_for the *i*-th strict set. The statistic for the given gene set is the minimum p-value over all the strict sets, i.e.

$$P_{min}=\overset{s}{\underset{i=c}{\min}}p_{i}.$$

### Permutation test

We employ the algorithm in [41] to assess the significance of the given gene set. The algorithm permutes the phenotypic data *C *= (*c*_1_, *c*_2_,⋯, *c*_*m*_) for *N *times and keep the gene expression data intact. After each permutation, the association strength measures are re-calculated using the permuted phenotypic data and the "strict subsets" are re-defined. Suppose that the statistics $T_{i}^{\left(n\right)}$ for the *n*-th permutation and the *i*-th strict subset are organized as

$$\left(\begin{array}{cccc} T_{c}^{\left(0\right)} & T_{c+1}^{\left(0\right)} & \cdots & T_{s}^{\left(0\right)}\\ T_{c}^{\left(1\right)} & T_{c+1}^{\left(1\right)} & \cdots & T_{s}^{\left(1\right)}\\ \cdots & \cdots & \cdots & \cdots \\ T_{c}^{\left(N\right)} & T_{c+1}^{\left(N\right)} & \cdots & T_{s}^{\left(N\right)} \end{array}\right),$$

where *c *is the minimum number of genes in the strict sets. Here we take the observed data as the permutation 0.

Based on the data matrix, we calculate the raw p-value for the *i*-th strict set of the observed data as

$$p_{i}=p_{i}^{\left(0\right)}=\frac{\#\{n>0:T_{i}^{\left(n\right)}\geq T_{i}^{\left(0\right)}\}}{N}.$$

The testing statistic for the gene set is $P_{min}=P_{min}^{\left(0\right)}=\min_{i=c}^{s}p_{i}^{\left(0\right)}$.

To estimate the distribution of *P*_*min *_under the null hypothesis, we replace the observed "strict subset" statistics with the permuted ones. The permuted raw p-values for the *n*-th permutation and *i*-th strict subset are calculated by

$$p_{i}^{\left(n\right)}=\frac{\#\{l\geq 0,l\neq n:T_{i}^{\left(l\right)}\geq T_{i}^{\left(n\right)}\}}{N}.$$

The minimum p-value $P_{min}^{\left(n\right)}=\min_{i=c}^{s}p_{i}^{\left(n\right)}$ is taken as one of the random sample from the distribution of *P*_*min *_under the null hypothesis. Finally the significance of the gene set is calculated as

$$p-value=\frac{\#\{n\geq 1:P_{min}^{\left(n\right)}\leq P_{min}^{\left(0\right)}\}}{N}$$

### Multiple testing correction for multiple gene sets

In a typical situation, there will be multiple gene sets to be analyzed. After we assess the significance level for each of them according to the procedure described above, q-values of all the given gene sets are calculated using the QVALUE R package [34] for multiple testing correction.

## Authors' contributions

XY developed and implemented the algorithm and FS provided the original idea. Both XY and FS contributed to the writing of the manuscript. XY and FS read and approved the final manuscript.

## Supplementary Material

Additional file 1 **Supplementary Materials**. This file contains the descriptions of the simulations that show the ROC curves and the robustness of Sub-GSE. Click here for file
