# Supplementary material for: Testing gene set enrichment for subset of genes: Sub-GSE
Source: BMC Bioinformatics. 2008 Sep 2;9:362. doi: 10.1186/1471-2105-9-362 (PMC2543030; doi:10.1186/1471-2105-9-362)
Supplement: Additional file 1 — Supplementary Materials. This file contains the descriptions of the simulations that show the ROC curves and the robustness of Sub-GSE. [file 1471-2105-9-362-S1.pdf]

Supplementary Materials

ROC curves for Sub-GSE

The number of relevant gene sets is one and two in simulation study 1 and 2 respectively which is too small to get a smooth ROC curve. Therefore, we did another simulation which is very similar to simulation 1 to show the sensitivity and specificity of Sub-GSE. The simulation is implemented according to the following steps:

1. Simulate 2 gene sets, each of which has 20 genes inside. There are no common genes in the two gene sets;
2. The gene expression levels of the 40 genes are generated from a multivariate normal distribution. The mean and the variance for each gene is 0 and 1. In each gene set, certain percentage of genes (PCG=10%, 20%,..., 90%) are correlated. The correlation coefficient between them varies from 0.1 to 0.9. The correlation coefficient between any other gene pair is 0.
3. For any given PCG and the correlation coefficient, simulate 1000 gene expression data sets for the 40 genes. For each simulation, randomly select one gene that is correlated with some other genes in the corresponding gene set. Take the gene expression profile of this gene as the phenotypic data and remove them from the gene expression data.

The simulation generates 1000 gene expression data sets as well as the corresponding phenotypic data for each given PCG (10%, 20%, ..., 90%) and correlation coefficient (0.1,0.2,...,0.9). We apply Sub-GSE to each data set and calculate the p-values for the two gene set. The result of the 1000 data sets for given PCG and correlation coefficient are pooled to draw the ROC curves. Different thresholds of the p-values are set and the corresponding sensitivity and specificity rates are calculated by comparing the gene sets that have p-values smaller than the threshold and those from which the corresponding phenotypic data is from. The ROC curves are shown in Figure 1:

According to Figure 1, once the PCG is larger than 0.3, the AUC score will be as high as 0.7 even for the smallest correlation coefficient. When PCG is smaller than 0.3, the AUC score can still be larger than 0.5.

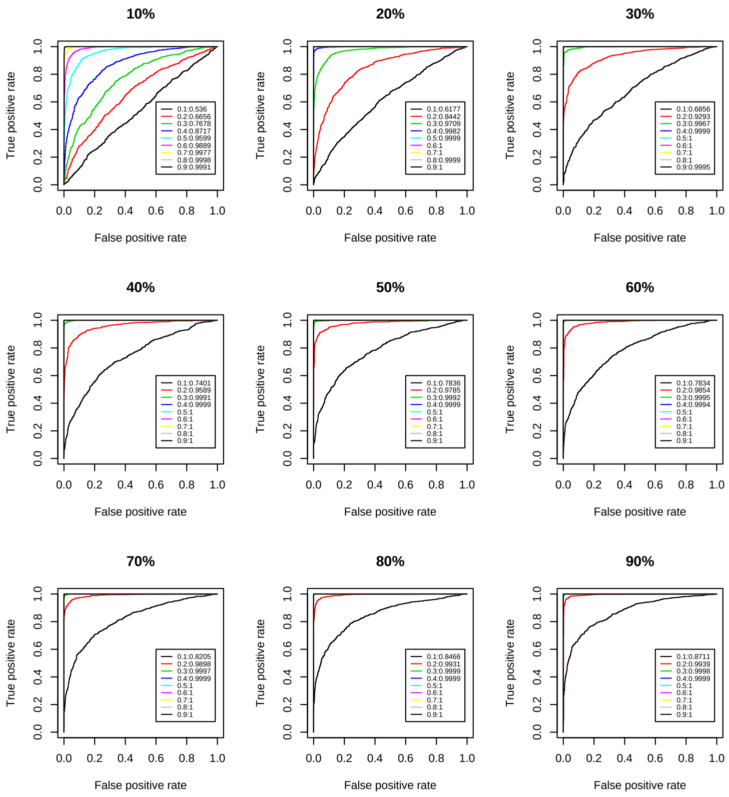

Figure 1: The ROC curves for Sub-GSE.
